# Long-Term Clinical Outcomes of Patients with Chronic Obstructive Pulmonary Disease with Sarcopenia

**DOI:** 10.3390/life13081628

**Published:** 2023-07-26

**Authors:** Yong Jun Choi, Taehee Kim, Hye Jung Park, Jae Hwa Cho, Min Kwang Byun

**Affiliations:** Department of Internal Medicine, Gangnam Severance Hospital, Yonsei University College of Medicine, Seoul 06273, Republic of Korea; cyj0717@yuhs.ac (Y.J.C.); sosom02@naver.com (T.K.); craft7820@yuhs.ac (H.J.P.); jhcho66@yuhs.ac (J.H.C.)

**Keywords:** sarcopenia, chronic obstructive pulmonary disease, mortality, exacerbation

## Abstract

Background and objective: Sarcopenia with muscle wasting and weakness is a common occurrence among patients with chronic obstructive pulmonary disease (COPD). We aimed to evaluate the clinical outcomes of sarcopenia in patients with COPD. Methods: We reviewed the electronic medical records of 71 patients with COPD between 1 January 2012, and 31 December 2018. We longitudinally analyzed clinical outcomes in patients with COPD with and without sarcopenia. Results: Compared to the non-sarcopenia group COPD, the sarcopenia group showed a higher rate of acute exacerbation events of COPD (AE COPD, 84.6% vs. 31.0%, *p* = 0.001), all-cause mortality (30.8% vs. 5.2%, *p* = 0.022), and pneumonia occurrence per year (median [first quartile–third quartile]; 0.2 [0.0–1.6] vs. 0.0 [0.0–0.2], *p* = 0.025). Sarcopenia was an independent risk factor for AE COPD in Cox regression analysis (hazard ratio, 5.982; 95% confidence interval, 1.576–22.704). Hand grip strength was associated with the COPD Assessment Test (CAT) score and annual Charlson’s comorbidity index score change. Total skeletal muscle mass index (SMMI) was associated with the modified medical research council dyspnea scale score, CAT score, body mass index, airflow obstruction, dyspnea, and exercise (BODE) index, and alanine transaminase. Trunk SMMI was significantly associated with AE COPD, while appendicular SMMI was associated with BODE index and annual intensive care unit admissions for AE COPD. Conclusions: Sarcopenia is associated with clinical prognosis, pneumonia occurrence, and the acute exacerbation of COPD requiring intensive care in patients with COPD. Therefore, it is important to carefully monitor sarcopenia development as well as recommend appropriate exercise and nutritional supplementation in patients with COPD.

## 1. Introduction

Chronic obstructive pulmonary disease (COPD) is a progressive and persistent airway disease with an enormous socioeconomic burden worldwide. COPD is associated with multi-morbid conditions, including cardiovascular disease, osteoporosis, muscle weakness, depression, and lung cancer [1]. Further, COPD is associated with loss of muscle mass, especially in patients with moderate-to-severe COPD and acute COPD exacerbations [2,3,4,5].

Sarcopenia, a progressive and systemic skeletal muscle disorder, is closely associated with an elevated risk of detrimental outcomes, including falls, fractures, physical disability, and mortality [6,7]. According to the European Working Group on Sarcopenia in Older People (EWGSOP2) guidelines, sarcopenia is diagnosed based on two main criteria: low muscle strength and low muscle quantity or quality. Additionally, when low physical performance is present in conjunction with these criteria, it is defined as severe sarcopenia [7]. The reported prevalence of sarcopenia is 10–27% in the general population [8]. Similarly, the prevalence of sarcopenia among patients with COPD ranges from 8% in population-based studies to 21% in clinic-based studies [9].

Sarcopenia has been reported to result in worsening clinical outcomes, forced expiratory volume in the first second (FEV_1_), and reduced exercise capacity and quality of life [10]. In particular, the acute exacerbation of COPD (AE COPD) and sarcopenia interact and adversely affect the prognosis of COPD [11]. However, data on the long-term clinical outcomes of patients with COPD who also have sarcopenia are still insufficient.

Based on the European Working Group on Sarcopenia in Older People (EWGSOP) criteria, we previously reported that the prevalence of sarcopenia among patients with COPD was 25% [12]. Moreover, sarcopenia was associated with respiratory symptoms and exercise tolerance in stable patients with COPD. However, this was a cross-sectional analysis, which limits the clinical interpretation of the findings. In addition, the Asian Working Group for Sarcopenia (AWGS) was updated in 2019 and can be applied to Asians [13].

We hypothesize that sarcopenia not only increases the risk of AE COPD but also correlates with worsening symptoms and an overall poor prognosis. Therefore, our objective was to assess the extended clinical outcomes of COPD patients with sarcopenia, as defined by the AWGS 2019 criteria, utilizing the cohort derived from the preceding cross-sectional study [12].

## 2. Materials and Methods

### 2.1. Study Design and Patients

**Figure 1:**
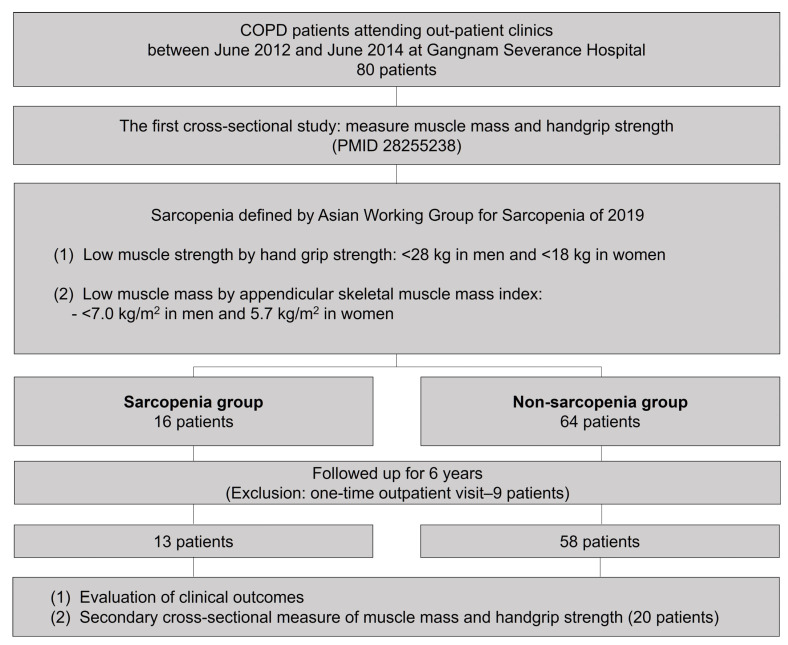
Study flow.

In our previous cross-sectional study, we enrolled eligible patients who provided written informed consent (Figure 1) [12]. The inclusion criteria were age >40 years, COPD diagnosis according to the Global Initiative for Chronic Obstructive Lung Disease, and outpatient visits between June 2012 and June 2014 to Gangnam Severance Hospital. We excluded patients with active lung disease, bronchial asthma, lung resection or transplantation, severe cardiovascular disease, COPD exacerbation within the last month, severe dyspnea impeding the six-minute walk distance test, lower-leg trauma, severe muscle weakness, and an inability to comprehend the informed consent documents. Patients were followed up at the outpatient clinic. We retrospectively reviewed their electronic medical records until 31 December 2018. Further, consenting patients were included in a secondary cross-sectional analysis of muscle mass measurements.

### 2.2. Measurement of Muscle Mass and Strength

Muscle mass was quantified using bioelectrical impedance analysis (BIA; Body Composition Analysis 1000; MediGate, Seoul, South Korea) in the first cross-sectional analysis and dual-energy X-ray absorptiometry (DEXA; Horizon W, Hologic Inc., Bedford, MA, USA), which is the gold-standard technique for molecular-level analysis of body composition [14], in the second cross-sectional analysis. The fat mass index (FMI) and fat-free mass index (FFMI) were calculated as the fat mass and fat-free mass (FFM), respectively, divided by the square of the patient’s height (kg/height^2^). The skeletal muscle mass index (SMMI) was calculated as skeletal muscle mass (SMM) divided by the square of the patient’s height (kg/height^2^). The appendicular and truncal SMMI (ASMI and TSMI, respectively) reflected the muscle mass of the limbs and trunk, respectively.

Handgrip strength (HGS) was measured using a Jamar handheld dynamometer (Patterson Medical, Warrenville, IL, USA), which has validated reliability in community-dwelling older adults. Participants were seated with the shoulders at 0° adduction and neutral rotation, elbows at 90° flexion, and forearms in a neutral position. The participants performed three trials with 1 min rest intervals, and the average values were recorded.

### 2.3. Definition of Sarcopenia

Sarcopenia was confirmed by the presence of both low muscle strength and low muscle mass based on the AWGS 2019 guidelines. Low muscle mass was defined as an appendicular skeletal muscle mass index (ASMI) by BIA of <7.0 kg/m^2^ in men and 5.7 kg/m^2^ in women. Low muscle strength was defined by HGS values of <28 kg in men and <18 kg in women.

### 2.4. Data Collection

Demographic characteristics included age, sex, height (cm), weight (kg), and body mass index (BMI; kg/m^2^). Comorbidities were analyzed using Charlson’s comorbidity index (CCI). FEV_1_, forced vital capacity (FVC), and forced mid-expiratory flow (FEF_25–75%_) were measured using spirometry (Vmax 229; SensorMedics, Yorba Linda, CA, USA), and converted to z-scores of the Global Lung Function Initiative 2012 (GLI 2012). Hemoglobin, aspartate aminotransferase (AST), alanine transaminase (ALT), blood urea nitrogen, creatinine, and C-reactive protein were measured at baseline and during follow-up visits. Interleukin-6 and high-sensitivity tumor necrosis factor-alpha (hsTNF-α) were measured in the second cross-sectional analysis.

### 2.5. Clinical Outcomes and Symptoms

The primary endpoint was the occurrence of AE COPD, while secondary endpoints included all-cause mortality, pneumonia development, outpatient/emergency room visits, and admissions to the general ward or intensive care unit (ICU). COPD symptoms were evaluated using the Korean version of the COPD Assessment Test (CAT) questionnaire (GlaxoSmithKline, London, UK) and the modified Medical Research Council (mMRC) dyspnea scale. We evaluated the BMI, degree of airflow obstruction, dyspnea, and exercise capacity using the BMI, airflow obstruction, dyspnea, and exercise (BODE) index [15].

## 3. Ethics

This study was approved by the Institutional Review Board (IRB) of Gangnam Severance Hospital (number: 3-2018-0109). Participants provided written informed consent to participate in the second cross-sectional study. Data were collected in accordance with the amended Declaration of Helsinki. All data for the retrospective analysis of clinical outcomes were fully anonymized before being accessed, and the ethics committee waived the requirement for written informed consent because of the retrospective nature of this study.

## 4. Statistical Analysis

Between-group comparisons of categorical variables were performed using the chi-square test or Fisher’s exact test. Based on the Shapiro–Wilk test, parametric and non-parametric continuous variables were compared using an independent two-sample t-test and Kruskal–Wallis rank sum test, respectively. Between-group comparisons of temporal changes in spirometry findings were performed using two-way repeated measures analysis of variance (RM-ANOVA). Correlations between variables were analyzed using Pearson’s correlation analysis. Statistical significance was set at *p* < 0.05.

Statistical analyses were conducted using R (version 4.0.2; R Foundation for Statistical Computing, Vienna, Austria) software. Cox regression analysis was performed using “survival” R package. In the multivariate model, age, sex, CCI, and variables with *p* < 0.100 in the univariate Cox regression analysis were included. Multicollinearity was assessed using the variance inflation factor (VIF), and a VIF of 5 or greater was considered to indicate the presence of multicollinearity. Pearson’s correlation analysis and RM-ANOVA were performed using the “stats” and “rstatix” R packages, respectively. Spirometry values were converted to z-score by reference values of GLI 2012 using the “rspiro” R package. Kaplan–Meier analysis was performed using the “survminer” R package.

## 5. Results

Baseline characteristics

Among 71 patients with COPD, there were 13 and 58 patients with and without sarcopenia, respectively (Table 1). There were no significant between-group differences in age, sex, height, CCI score, waist–hip ratio, smoking history, and treatment of COPD. Body weight and BMI were lower in the sarcopenia group than in the non-sarcopenia group (median [first quartile; third quartile]; 56.0 [50.0; 61.7] vs. 63.9 [59.0; 70.0], *p* = 0.005; and 21.5 [20.1;22.5] vs. 23.2 [21.5; 25.7], *p* = 0.003; respectively). SMMI, FFM, and FMMI were lower in the sarcopenia group than in the non-sarcopenia group (mean ± standard deviation; 8.6 ± 0.7 vs. 9.7 ± 0.9, *p* < 0.001; 43.7 ± 4.6 vs. 48.9 ± 6.8, *p* = 0.011; and 16.1 ± 1.0 vs. 17.6 ± 1.2, *p* < 0.001; respectively). There were no significant between-group differences in fat mass and FMI (13.0 ± 3.7 vs. 16.2 ± 5.6, *p* = 0.054 and 4.8 ± 1.5 vs. 5.9 ± 2.0, *p* = 0.086; respectively). HGS was lower in the sarcopenia group than in the non-sarcopenia group (26.3 [24.7; 26.3] vs. 33.7 [29.0; 39.3], *p* < 0.001). There were no significant between-group differences in baseline spirometry findings. Regarding baseline laboratory data, only ALT (IU/L) levels were higher in the sarcopenia group than in the non-sarcopenia group (14.0 [11.0; 18.0] vs. 19.0 [14.0; 23.0], *p* = 0.030).

## 6. Clinical Outcomes and Prognosis of Patients with COPD with Sarcopenia

Throughout the observation period, the sarcopenia group experienced a higher incidence (advent of events) and frequency (events per year) of AE COPD than non-sarcopenia group (84.6% vs. 31.0%, *p* = 0.001 and 0.6 [0.3; 1.1] vs. 0.0 [0.0; 0.2], *p* = 0.001, respectively; Table 2). Furthermore, the experience and frequency of admissions for COPD (84.6% vs. 27.6%, *p* < 0.001 and 0.6 [0.3; 1.1] vs. 0.0 [0.0; 0.2], *p* < 0.001; respectively), and experience and frequency of ICU admissions for AE COPD (30.8 vs. 3.4%, *p* = 0.008 and 0.0 [0.0; 0.2] vs. 0.0 [0.0; 0.0], *p* = 0.001; respectively), all-cause admissions (92.3% vs. 55.2%, *p* = 0.029), ICU admissions (38.5% vs. 5.2%, *p* = 0.003), frequency of all-cause admission (0.5 [0.2; 2.2] vs. 0.2 [0.0; 0.5], *p* = 0.025), and frequency of ICU admission (0.0 [0.0; 0.2] vs. 0.0 [0.0; 0.0], *p* = 0.001) were all higher in the sarcopenia group. The sarcopenia group had a higher all-cause mortality rate (30.8% vs. 5.2%, *p* = 0.022).

The sarcopenia group had a significantly higher frequency of annual pneumonia (0.2 [0.0; 1.6] vs. 0.0 [0.0; 0.2], *p* = 0.025), but their experience of pneumonia was not higher than the non-sarcopenia group (53.8% vs. 31.0%, *p* = 0.217). There were no between-group differences in annual changes in the z-score of FEV_1_, FVC, FEV_1_/FVC, and FEF_25–75%_ (*p* = 0.960, *p* = 1.000, *p* = 0.990, and *p* = 0.980, respectively; Figure S1).

**Figure 2:**
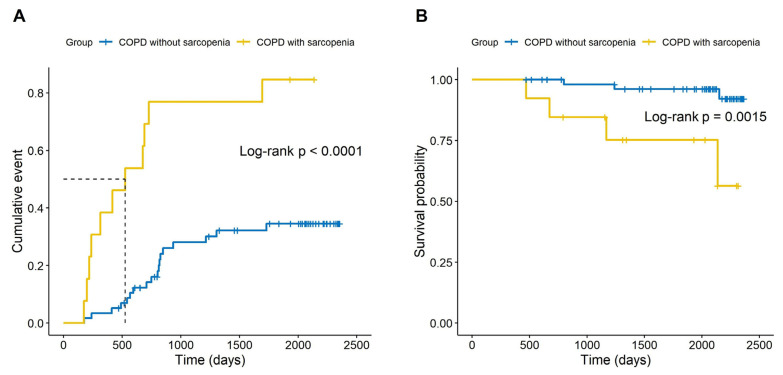
Cumulative acute exacerbation events and all-cause mortality of chronic obstructive pulmonary disease. (**A**) Acute exacerbation of chronic obstructive pulmonary disease. (**B**) All-cause mortality in patients with chronic obstructive pulmonary disease.

In the Kaplan–Meier analysis, cumulative AE COPD and all-cause mortality were significantly higher in COPD with sarcopenia than COPD without sarcopenia (median follow-up 1305 and 2075 days, Figure 2A and Figure 2B, respectively).

In the Cox regression analysis for hazard ratios of AE COPD, statistically significant variables in the univariate analysis were pneumonia development per year (hazard ratio (HR) 2.430; 95% confidence interval [CI], 1.607–3.676, Table 3), fat-free mass index (HR 0.728; 95% CI, 0.541–0.979), ASMI (HR, 0.533; 95% CI, 0.298–0.952), hand grip strength (HR, 0.951; 95% CI, 0.917–0.988), and sarcopenia (HR, 5.311; 95% CI, 2.479–11.379). In the multivariate analysis, pneumonia development per year (HR, 2.094; 95% CI, 1.271–3.449), FEV_1_/FVC z-score (HR, 0.607; 95% CI, 0.427–0.864), and sarcopenia (HR, 5.982; 95% CI, 1.576–22.704) were identified as significant factors for AE COPD.

## 7. Correlations of HGS, SMMI, TSMI, and ASMI with Clinical Parameters in the Secondary Cross-Sectional Analysis

A total of 20 patients participated in secondary cross-sectional analysis (Supplementary Table S1). HGS was negatively correlated with age, CAT score change, and annual CCI score change (correlation coefficient [r] = −0.490, *p* = 0.028; r = −0.468, *p* = 0.038; and r = −0.473, *p* = 0.035; respectively; Table 4); moreover, it was positively correlated with BMI, total SMMI, and ASMI (r = 0.515, *p* = 0.020; r = 0.641, *p* = 0.003; and r = 0.645, *p* = 0.004, respectively).

SMMI was negatively correlated with mMRC, CAT score, and BODE index (r = −0.515, *p* = 0.024; r = −0.482, *p* = 0.050; and r = −0.638, *p* = 0.003, respectively); further, it was positively correlated with BMI, FFMI, TSMI, ASMI, HGS, AST, and ALT (r = 0.852, *p* < 0.001; r = 0.992, *p* < 0.001; r = 0.763, *p* < 0.001; r = 0.895, *p* < 0.001; r = 0.641, *p* = 0.003; r = 0.543, *p* = 0.016; and r = 0.466, *p* = 0.044, respectively).

TSMI was negatively correlated with experience of AE COPD (r = −0.562, *p* = 0.019) and positively correlated with BMI, FFMI, SMMI, and AST (r = 0.483, *p* = 0.050; r = 0.715, *p* = 0.001; r = 0.763, *p* < 0.001; and r = 0.563, *p* = 0.019, respectively).

ASMI was negatively correlated with BODE index and ICU admissions per year (r = −0.648, *p* = 0.004 and r = −0.479, *p* = 0.044, respectively); furthermore, it was positively correlated with BMI, FFMI, FMI, SMMI, and HGS (r = 0.890, *p* < 0.001; r = 0.716, *p* = 0.001; r = 0.611, *p* = 0.009; r = 0.895, *p* < 0.001; and r = 0.645, *p* = 0.004, respectively).

## 8. Discussion

Sarcopenia was associated with all-cause mortality, COPD exacerbation, and pneumonia development in patients with COPD. These findings suggest the need for early sarcopenia identification; in addition, nutritional supplementation and exercise are recommended for patients with COPD.

According to the EWGSOP2 and AWGS 2019 guidelines, low muscle strength is a characteristic feature of sarcopenia. Muscle strength is measured using the HGS and chair stand tests, while muscle quantity/quality can be measured using DEXA or BIA. However, there is no specific screening protocol for sarcopenia in patients with COPD, especially Asian patients. Accordingly, there is a need to establish a specified cutoff value for Asian patients and determine the optimal period of sarcopenia screening in patients with COPD.

We designed the current study as a follow-up of our prior study [12]. For the definition of sarcopenia in the previous study, low muscle mass was defined as a skeletal muscle mass index (SMMI) at least two standard deviations below normal sex-specific means in young persons, and low muscle strength was defined as hand grip strength (HGS) values of ≤30 kg in men and ≤20 kg in women, according to the recommendation of the European Working Group on Sarcopenia in Older People (EWGSOP). However, in early 2018, the Working Group updated the original definition of sarcopenia with the EWGSOP2 definition, with a focus on low muscle strength as a key characteristic of sarcopenia. We assessed the clinical significance of HGS in patients with COPD. We observed a negative correlation between CAT score, annual CCI score changes and HGS. Similarly, Martinez et al. reported that a higher handgrip strength (HGS) is associated with a lower frequency of exacerbations in patients with COPD [16]. The identification of handgrip weakness is a straightforward measure that not only provides prognostic information but also complements known predictors such as the BODE index and BMI in assessments of patients with COPD [17]. Therefore, periodic HGS evaluation can be useful for the evaluation of sarcopenia and sarcopenia-related outcomes in COPD patients; however, further research is required.

The main parameter for muscle mass measurements in previous studies is ASMI [7,9,18,19]. We analyzed the association between muscle mass in each body part (total SMMI, TSMI, and ASMI) and clinical outcomes. There were strong correlations among the muscle mass parameters; however, they showed different associations with clinical outcomes. Specifically, TSMI showed a stronger association with COPD symptoms than ASMI, while ASMI showed a stronger association with BODE index and ICU admissions for AE COPD than TSMI. Additionally, total SMMI tended to better reflect various clinical outcomes than TSMI or ASMI. Therefore, total SMMI may be a better evaluation index for muscle mass in patients with COPD than ASMI; however, we conducted the study with a small sample size and, as this was a secondary study utilizing participants from the previous research, we did not perform a proper sample size evaluation. Therefore, further research with an appropriate sample size assessment is warranted.

Sarcopenia is correlated with mortality in the general population. Bachettini et al. reported that severe sarcopenia according to EWGSOP2 increased mortality in older people (hazard ratio 4.11, 95% confidence interval 1.88–9.00) [20]. Similarly, Miguel et al. showed a synergistic effect of sarcopenia and COPD on mortality in older patients with COPD [21]. Although we included a small sample size, the sarcopenia group showed a higher all-cause mortality rate than the non-sarcopenia group. Sarcopenia and HGS are associated with the frequency of AE COPD [2,22,23]. Perrot et al. reported that sarcopenia was highly prevalent among patients with COPD during an acute exacerbation (48%) and after recovery (30%) [2]. Lee et al. reported that HGS at admission for AE COPD was negatively associated with risk of exacerbation in the following year [23]. In our analysis, muscle mass and HGS were significant risk factors for AE COPD in the univariate analysis; however, their statistical significance diminished in the multivariate analysis. Interestingly, sarcopenia, a variable that considers both muscle mass and HGS, showed statistically significant results. This suggests the importance of evaluating sarcopenia in COPD patients by considering both muscle mass and hand grip strength, rather than considering each separately.

Sarcopenia is a risk factor for pneumonia in older people, and there is a relationship between respiratory muscle strength and pneumonia [24]. We observed no significant relationship between sarcopenia and experience of pneumonia; however, annual pneumonia development was higher in the sarcopenia group than in the non-sarcopenia group. Muscle strength and muscle mass were not significantly associated with annual pneumonia development, which could be attributed to our small sample size; further large-scale studies are warranted.

We found that ALT was significantly associated with sarcopenia and positively correlated with muscle mass. Several studies have reported a relationship between ALT and sarcopenia [25,26,27]. ALT levels are commonly used to evaluate liver disease; however, they can reflect skeletal muscle volume as ALT is also distributed in the heart, muscles, and kidney [28]. Accordingly, low ALT levels are associated with COPD development, acute exacerbations, and mortality [28,29]. Lasman et al. reported that low ALT levels are associated with mortality in patients with COPD with a history of exacerbation [29]. Taken together, these findings demonstrate the clinical utility of measuring ALT levels in patients with COPD.

In contrast to the results of previous studies [30,31], there were no significant between-group differences in baseline and annual changes in spirometry findings in the current study. Nathalie et al. described that FEV1 decreased more in patients with COPD who also had sarcopenia than in those without sarcopenia [30]. Park et al. reported that a decrease in MMI was significantly associated with accelerated FEV1 decline in men [31]. In the current study, we included only 71 patients with COPD and a follow-up period of less than 5 years, retrospectively. The small number of patients and short follow-up duration may have affected the result.

Recent studies also describe that chronic low-grade inflammation may play an important role in the development of sarcopenia. Byun et al. [12] and Gao et al. [32] reported that inflammatory markers, such as IL-6, hs-TNFα, and resistin, are associated with sarcopenia. These associations have been debated in several studies. Tang et al. did not support the utility of the inflammatory indexes, such as neutrophil, lymphocyte, monocyte, and platelet counts, and C-reactive protein (CRP) as biomarkers of sarcopenia [33]. Kamper et al. also reported that only high levels of hsCRP, but not TNF-α, IL-4, and IFN-γ, were weakly associated with low muscle mass [34]. The current study also did not show a significant association between the inflammatory markers CRP, IL-6, and hsTNF-α. Because inflammatory markers are affected by various factors, such as infections, diseases, and sarcopenia, different results can be reported depending on the patient’s accompanying conditions. Furthermore, this also suggests that chronic low-grade inflammation is not the main cause of sarcopenia.

A strength of our study is its long-term follow-up period (6 years) of patients with COPD and sarcopenia, which revealed a correlation between sarcopenia and all-cause mortality, COPD exacerbation, and pneumonia development. However, this study has several limitations. First, the sample size was small, which might have contributed to the lack of statistical significance for some findings. In addition, we did not conduct sample size calculations for the comparison of each variable. Consequently, this may have resulted in inadequate sample sizes for certain variables, potentially leading to an inability to achieve statistical significance. Second, clinical outcomes were evaluated through a retrospective review of electronic medical records, which could have resulted in biases. Third, the use of BIA measurements in the first cross-sectional study and DEXA measurements in the second cross-sectional study presents a limitation to the accurate evaluation of changes that occurred over the observation period.

In conclusion, sarcopenia is a common comorbidity that negatively affects clinical outcomes in patients with COPD. Specifically, sarcopenia is positively associated with all-cause mortality, acute exacerbations, and pneumonia occurrence in patients with COPD. These findings suggest the need for early sarcopenia identification, as well as recommending nutritional supplementation and exercise for patients with COPD. Moreover, protocols and guidelines for screening sarcopenia in patients with COPD may be required.

## Figures and Tables

**Table 1:** Baseline characteristics.

Variables	Total	Non-Sarcopenia	Sarcopenia	*p*-Value
	(N = 71)	(N = 58)	(N = 13)	
Age (years)	68.3 ± 9.2	67.3 ± 9.1	72.4 ± 9.2	0.076
Sex (Male)	61 (85.9%)	48 (84.2%)	13 (92.9%)	0.770
Height (cm)	166.1 ± 6.7	166.4 ± 6.7	164.6 ± 6.7	0.396
Weight (kg)	62.0 (57.0; 70.0)	63.9 (59.0; 70.0)	56.0 (50.0; 61.7)	0.005 *
BMI (kg/m^2^)	23.0 (21.2; 25.1)	23.2 (21.5; 25.7)	21.5 (20.1; 22.5)	0.003 *
CCI score (points)	1.0 (1.0; 3.0)	1.0 (1.0; 3.0)	1.0 (1.0; 3.0)	1.000
GOLD classification (2022)				0.965
A	17 (23.9%)	14 (24.1%)	3 (23.1%)	
B	40 (56.3%)	32 (55.2%)	8 (61.5%)	
C	8 (11.3%)	7 (12.1%)	1 (7.7%)	
D	6 (8.5%)	5 (8.6%)	1 (7.7%)	
Smoking history				0.686
Never smoker	29 (40.8%)	25 (43.1%)	4 (30.8%)	
Ex-smoker	25 (35.2%)	20 (34.5%)	5 (38.5%)	
Current smoker	17 (23.9%)	13 (22.4%)	4 (30.8%)	
Treatment for COPD				
Beta agonists	58 (81.7%)	47 (81.0%)	11 (84.6%)	1.000
Anticholinergics	61 (85.9%)	50 (86.2%)	11 (84.6%)	1.000
Methylxanthines	23 (32.4%)	16 (27.6%)	7 (53.8%)	0.133
Corticosteroids	43 (60.6%)	32 (55.2%)	11 (84.6%)	0.099
Waist hip ratio	0.9 ± 0.0	0.9 ± 0.1	0.9 ± 0.0	0.525
SMMI (kg/m^2^)	9.5 ± 0.9	9.7 ± 0.9	8.6 ± 0.7	<0.001 *
TSMI	7.6 ± 0.7	7.8 ± 0.7	7.0 ± 0.6	0.001 *
ASMI	7.0 ± 0.7	7.1 ± 0.6	6.4 ± 0.5	<0.001 *
Upper extremities	1.8 (1.6; 2.0)	1.9 (1.7; 2.0)	1.7 (1.5; 1.7)	0.002 *
Lower extremities	5.2 ± 0.5	5.2 ± 0.4	4.8 ± 0.4	0.001 *
Fat mass (kg)	15.6 ± 5.4	16.2 ± 5.6	13.0 ± 3.7	0.054
Fat mass index (kg/m^2^)	5.7 ± 2.0	5.9 ± 2.0	4.8 ± 1.5	0.086
Fat-free mass (kg)	47.9 ± 6.7	48.9 ± 6.8	43.7 ± 4.6	0.011 *
Fat-free mass index (kg/m2)	17.3 ± 1.3	17.6 ± 1.2	16.1 ± 1.0	<0.001 *
Hand grip strength (kg)	30.7 (26.1; 38.0)	33.7 (29.0; 39.3)	26.3 (24.7; 26.3)	<0.001 *
Baseline spirometer				
FVC (liters)	3.1 (2.4; 3.5)	3.0 (2.4; 3.5)	3.4 (2.4; 3.8)	0.405
FVC (z-score)	−1.8 (−2.6; −0.0)	−1.9 (−2.8; −0.5)	0.2 (−1.8; 0.4)	0.062
FEV1 (liters)	1.5 ± 0.4	1.5± 0.4	1.4 ± 0.5	0.405
FEV1 (z-score)	−3.6 (−4.6; −2.7)	−3.6 (−4.6; −2.7)	−4.0 (−4.6; −2.9)	0.888
FEV1/FVC (%)	51.0 ± 13.2	52.5 ± 13.4	44.5 ± 10.2	0.049 *
FEV1/FVC (z-score)	−3.8 (−4.9; −2.8)	−3.7 (−4.7; −2.4)	−4.6 (−5.1; −3.6)	0.100
FEF_25–75%_ (liter/sec)	0.5 (0.3; 0.8)	0.5 (0.3; 0.8)	0.4 (0.3; 0.5)	0.112
FEF_25–75%_ (z-score)	−2.9 ± 0.9	−2.8 ± 0.9	−3.1 ± 1.1	0.350
Baseline laboratory data				
Hemoglobin (g/dL)	14.4 (13.0; 14.9)	14.4 (12.9; 14.9)	14.4 (13.6; 15.0)	0.657
AST (IU/L)	20.0 (19.0; 25.0)	21.0 (19.0; 26.0)	20.0 (18.0; 21.0)	0.190
ALT (IU/L)	18.0 (13.0; 22.0)	19.0 (14.0; 23.0)	14.0 (11.0; 18.0)	0.030 *
BUN (mg/dL)	15.2 (13.1; 19.9)	15.8 (13.2; 15.9)	14.6 (12.3; 15.3)	0.388
Creatinine (mg/dL)	0.8 (0.7; 1.0)	0.8 (0.7; 1.0)	0.8 (0.7; 0.9)	0.326
CRP (mg/dL)	1.7 (0.7; 4.4)	2.0 (0.8; 4.5)	1.1 (0.3; 2.8)	0.135

Abbreviations: ALT, alanine transaminase; AST, aspartate aminotransferase; ASMI, appendicular muscle mass index; BMI, body mass index; BUN, blood urea nitrogen; CCI, Charlson’s comorbidity index; COPD, chronic obstructive pulmonary disease; CRP, C-reactive protein; FEF_25–75%_, forced mid-expiratory flow; FVC, forced vital capacity; FEV_1_, forced expiratory volume in the first second; N, number; SMMI, skeletal muscle mass index; TSMI, trunk muscle mass index. Data are presented as mean ± standard deviation for parametric continuous variables, median (first quartile; third quartile) for non-parametric continuous, and *n* (%) for categorical variables; * *p* < 0.05.

**Table 2:** Comparison of clinical outcomes between groups during the observational period.

Group	Total	Non-Sarcopenia	Sarcopenia	*p*-Value
	(N = 71)	(N = 58)	(N = 13)	
COPD AE	29 (40.8%)	18 (31.0%)	11 (84.6%)	0.001 *
ER visits for COPD AE	23 (32.4%)	16 (27.6%)	7 (53.8%)	0.133
Admission for COPD AE	27 (38.0%)	16 (27.6%)	11 (84.6%)	<0.001 *
ICU admissions for COPD AE	6 (8.5%)	2 (3.4%)	4 (30.8%)	0.008 *
COPD AE per year	0.0 (0.0; 0.5)	0.0 (0.0; 0.2)	0.6 (0.3; 1.1)	0.001 *
ER visits per year caused by COPD AE	0.0 (0.0; 0.3)	0.0 (0.0; 0.2)	0.2 (0.0; 1.1)	0.081
Admissions per year caused by COPD AE	0.0 (0.0; 0.4)	0.0 (0.0; 0.2)	0.6 (0.3; 1.1)	<0.001 *
ICU admissions per year caused by COPD AE	0.0 (0.0; 0.0)	0.0 (0.0; 0.0)	0.0 (0.0; 0.2)	0.001 *
All-cause mortality	7 (9.9%)	3 (5.2%)	4 (30.8%)	0.022 *
Pneumonia-associated mortality	4 (57.1%)	2 (66.7%)	2 (50.0%)	0.659
Malignancy-associated mortality	3 (42.9%)	1 (33.3%)	2 (50.0%)	0.659
Pneumonia development	25 (35.2%)	18 (31.0%)	7 (53.8%)	0.217
Pneumonia development per year	0.0 (0.0; 0.2)	0.0 (0.0; 0.2)	0.2 (0.0; 1.6)	0.025 *
Annual CCI score changes (points)	0.0 (0.0; 0.3)	0.0 (0.0; 0.3)	0.2 (0.0; 0.4)	0.170
Lung cancer development	7 (10.0%)	6 (10.5%)	1 (7.7%)	1.000
Visit OPD				
All departments per year	9.9 (5.7; 15.0)	10.0 (5.2; 14.7)	9.0 (8.2; 20.2)	0.471
Pulmonology per year	4.4 (2.9; 5.5)	4.2 (2.4; 5.4)	5.1 (3.9; 6.0)	0.226
All-cause ER visit	29 (40.8%)	22 (37.9%)	7 (53.8%)	0.457
All-cause ER visits per year	0.0 (0.0; 0.2)	0.0 (0.0; 0.2)	0.2 (0.0; 1.1)	0.127
All-cause admission	44 (62.0%)	32 (55.2%)	12 (92.3%)	0.029 *
All-cause admissions per year	0.2 (0.0; 0.5)	0.2 (0.0; 0.5)	0.5 (0.2; 2.2)	0.025 *
All-cause ICU admission	8 (11.3%)	3 (5.2%)	5 (38.5%)	0.003 *
All-cause ICU admission per year	0.0 (0.0; 0.0)	0.0 (0.0; 0.0)	0.0 (0.0; 0.2)	0.001 *

Abbreviations: N, number; COPD, chronic obstructive pulmonary disease; AE, acute exacerbation; ER, emergency room; ICU, intensive care unit; CCI, Charlson’s comorbidity index; OPD, outpatient department. Data are presented as median (first quartile; third quartile) for non-parametric continuous variables, and n (%) for categorical variables; * *p* < 0.05.

**Table 3:** Cox regression analysis for hazard ratio of COPD acute exacerbations.

	Univariate Analysis		Multivariate Analysis		
	Hazard Ratio (95% Confidence Interval)	*p*-Value	Hazard Ratio (95% Confidence Interval)	*p*-Value	VIF
Age (years)	1.048 (0.998–1.101)	0.059	1.033 (0.972–1.098)	0.296	1.582
Sex (vs. males)	1.234 (0.429–3.549)	0.697	4.055 (0.580–28.355)	0.158	3.291
CCI score (points)	0.953 (0.656–1.383)	0.799	1.103 (0.716–1.701)	0.656	1.137
BMI (kg/m^2^)	0.913 (0.804–1.036)	0.158			
Smoking history (vs. never-smoker)					
Ex-smoker	1.096 (0.465–2.583)	0.834			
Current smoker	1.284 (0.516–3.194)	0.590			
Pneumonia development per year	2.430 (1.607–3.676)	<0.001 *	2.094 (1.271–3.449)	0.004 *	1.334
Baseline spirometer					
FVC (z-score)	1.132 (0.962–1.332)	0.134			
FEV1 (z-score)	0.921 (0.691–1.227)	0.574			
FEV1/FVC (z-score)	0.784 (0.609–1.010)	0.060	0.607 (0.427–0.864)	0.006 *	1.531
FEF_25–75%_ (z-score)	0.836 (0.572–1.224)	0.358			
Fat free mass index (kg/m^2^)	0.728 (0.541–0.979)	0.036 *	Omitted due to multicollinearity		
Fat mass index (kg/m^2^)	0.991 (0.816–1.203)	0.927			
ASMI (kg/m^2^)	0.533 (0.298–0.952)	0.033 *	1.527 (0.662–3.519)	0.321	1.877
Hand grip strength (kg)	0.951 (0.917–0.988)	0.009 *	0.994 (0.914–1.080)	0.885	3.512
Baseline laboratory data					
Hemoglobin (g/dL)	0.900 (0.701–1.157)	0.412			
AST (IU/L)	0.973 (0.925–1.022)	0.275			
ALT (IU/L)	0.987 (0.946–1.030)	0.545			
BUN (mg/dL)	0.958 (0.885–1.039)	0.300			
Creatinine (mg/dL)	0.780 (0.252–2.420)	0.668			
CRP (mg/dL)	1.001 (0.983–1.019)	0.921			
Sarcopenia	5.311 (2.479–11.379)	<0.001 *	5.982 (1.576–22.704)	0.009 *	2.527

Abbreviations: ALT, alanine transaminase; AST, aspartate aminotransferase; ASMI, appendicular muscle mass index; BMI, body mass index; BUN, blood urea nitrogen; CCI, Charlson’s comorbidity index; COPD, chronic obstructive pulmonary disease; CRP, C-reactive protein; FEF_25–75%_, forced mid-expiratory flow; FVC, forced vital capacity; FEV_1_, forced expiratory volume in the first second; VIF, variance inflation factor. * *p* < 0.05.

**Table 4:** Correlations of HGS, SMMI, TSMI, and ASMI with clinical parameters in the secondary cross-sectional analysis.

	HGS		SMMI		Trunk SMMI		ASMI	
	Correlation Coefficient	*p*-Value	Correlation Coefficient	*p*-Value	Correlation Coefficient	*p*-Value	Correlation Coefficient	*p*-Value
Age (years)	−0.490	0.028 *	−0.260	0.282	−0.344	0.176	0.011	0.965
BMI (kg/m^2^)	0.515	0.020 *	0.852	<0.001 *	0.483	0.050 *	0.890	<0.001 *
FFMI (kg/m^2^)	0.431	0.058	0.992	<0.001 *	0.715	0.001 *	0.716	0.001 *
FMI (kg/m^2^)	0.275	0.255	0.449	0.054	0.101	0.699	0.611	0.009 *
Total SMMI (kg/m^2^)	0.641	0.003 *	NA	NA	0.763	<0.001 *	0.895	<0.001 *
TSMI (kg/m^2^)	0.348	0.171	0.763	<0.001 *	NA	NA	0.394	0.118
ASMI (kg/m^2^)	0.645	0.004 *	0.895	<0.001 *	0.394	0.118	NA	NA
Hand grip strength	NA	NA	0.641	0.003 *	0.348	0.171	0.645	0.004 *
Clinical parameters								
mMRC	−0.092	0.701	−0.515	0.024 *	−0.372	0.141	−0.434	0.072
CAT score	−0.468	0.038 *	−0.482	0.050 *	−0.455	0.066	−0.300	0.226
6MWD	0.390	0.099	0.381	0.119	0.396	0.116	0.269	0.281
BODE index	−0.244	0.300	−0.638	0.003 *	−0.319	0.212	−0.648	0.004 *
Annual CCI score changes (points)	−0.473	0.035 *	−0.098	0.690	0.080	0.761	−0.153	0.545
Pneumonia developments per year	0.101	0.671	−0.100	0.685	−0.200	0.442	0.010	0.968
COPD acute exacerbation	−0.232	0.325	−0.437	0.062	−0.562	0.019*	−0.220	0.381
Total counts per year	−0.142	0.550	−0.381	0.107	−0.435	0.081	−0.227	0.366
ER visits per year	−0.169	0.476	−0.398	0.092	−0.431	0.084	−0.254	0.310
Admissions per year	−0.081	0.735	−0.262	0.279	−0.311	0.225	−0.141	0.576
ICU admissions per year	−0.243	0.301	−0.427	0.068	−0.175	0.501	−0.479	0.044 *
Laboratory findings								
Hemoglobin (g/dL)	0.015	0.950	−0.068	0.781	−0.156	0.550	0.117	0.645
AST (IU/L)	0.029	0.904	0.543	0.016 *	0.563	0.019*	0.231	0.356
ALT (IU/L)	0.059	0.803	0.466	0.044 *	0.478	0.052	0.064	0.799
BUN (mg/dL)	−0.094	0.692	0.381	0.108	0.461	0.063	0.296	0.234
Creatinine (mg/dL)	−0.091	0.703	0.200	0.412	0.418	0.095	0.123	0.628
CRP (mg/dL)	0.160	0.501	0.097	0.692	0.197	0.449	−0.090	0.723
IL-6	−0.129	0.611	0.077	0.768	0.084	0.757	0.147	0.573
hsTNF-α	−0.174	0.464	−0.236	0.330	−0.281	0.275	−0.125	0.621

Abbreviations: BMI, body mass index; FFMI, fat-free mass index; FMI, fat mass index; SMMI, skeletal muscle mass index; TSMI, trunk muscle mass index; ASMI, appendicular muscle mass index; mMRC, modified medical research council dyspnea scale; CAT, chronic obstructive pulmonary disease assessment test; 6MWD, six-minute walk distance test; BODE, body mass index, airflow obstruction, dyspnea, and exercise index; CCI, Charlson’s comorbidity index; COPD, chronic obstructive pulmonary disease; ER, emergency room; ICU, intensive care unit; AST, aspartate aminotransferase; ALT, alanine transaminase; BUN, blood urea nitrogen; CRP, C-reactive protein; IL-6, interleukin-6; hsTNF-α, high-sensitivity tumor necrosis factor-alpha. * *p* < 0.05

## Data Availability

The data presented in this study are available on request from the corresponding author. The data are not publicly available due to no permission for data provision when IRB approval was obtained. Data can be made available from the first or corresponding author (contact: Yong Jun Choi or Min Kwang Byun, cyj0717@yuhs.ac or littmann@yuhs.ac, respectively) and IRB (gnocr@yuhs.ac) for re-searchers who meet the criteria for access to confidential data.

## Supplementary Materials

The following supporting information can be downloaded at: https://www.mdpi.com/article/10.3390/life13081628/s1, Table S1: Baseline characteristics of participants in secondary cross-sectional analysis; Figure S1: Annual changes in spirometry findings

## Abbreviations

AE COPD	acute exacerbation of chronic obstructive pulmonary disease
ALT	alanine transaminase
ASMI	appendicular skeletal muscle mass index
AST	aspartate aminotransferase
AWGS	Asian Working Group for Sarcopenia
BIA	bioelectrical impedance analysis
BMI	body mass index
BODE	body mass index, airflow obstruction, dyspnea, and exercise
CAT	Chronic Obstructive Pulmonary Disease Assessment Test
CCI	Charlson’s comorbidity index
COPD	chronic obstructive pulmonary disease
DEXA	dual-energy X-ray absorptiometry
EWGSOP	European Working Group on Sarcopenia in Older People
EWGSOP2	European Working Group on Sarcopenia in Older People of 2018
FEF_25–75%_	forced mid-expiratory flow
FEV_1_	Forced expiratory volume in the first second
FFM	fat-free mass
FFMI	fat-free mass index
FMI	fat mass index
FVC	forced vital capacity
GLI 2012	Global Lung Function Initiative 2012 (GLI 2012)
HGS	handgrip strength
ICU	intensive care unit
mMRC	modified Medical Research Council
SMM	skeletal muscle mass
SMMI	skeletal muscle mass index
RM-ANOVA	repeated measures analysis of variance
TSMI	truncal skeletal muscle mass index
