# Supplementary material for: Long-Term Clinical Outcomes of Patients with Chronic Obstructive Pulmonary Disease with Sarcopenia
Source: Life (Basel). 2023 Jul 26;13(8):1628. doi: 10.3390/life13081628 (PMC10455166; doi:10.3390/life13081628)

Supplementary data

Table S1. Baseline characteristics of participants in secondary cross-sectional analysis

Variables	Total (N=20)
Age (years)	65.6±9.0
Sex	
Male	20 (100.0%)
Height (cm)	167.9±6.0
Weight (kg)	64.9±8.9
BMI (kg/m^2)	23.3±2.7
CCI score (points)	
-1	14 (70.0%)
-2	1 (5.0%)
-3	3 (15.0%)
-4	1 (5.0%)
-5	1 (5.0%)
Waist hip ratio	0.9±0.0
SMMI (kg/m2)	9.8±0.9
TSMI	7.9±0.6
ASMI	7.2±0.6
Upper extremities	1.9±0.2
Lower extremities	5.3±0.5
Fat mass (kg)	15.6±4.8
Fat mass index (kg/m^2)	5.6±1.7
Fat free mass (kg)	50.4 [44.5; 55.3]
Fat free mass index (kg/m2)	17.8±1.2
Hand grip strength (kg)	36.7±6.6
Baseline spirometer	
FVC (z-score)	-0.8±2.8
FEV_1 (z-score)	-3.2 [-4.2; -2.6]
FEV_1/FVC (z-score)	-3.9 [-4.7; -2.1]
$FEF_{25\%–75\%}$ (z-score)	-2.9±1.1
Baseline laboratory data	
Hemoglobin (g/dL)	14.5±0.9
AST (IU/L)	24.4±4.6
ALT (IU/L)	23.9±6.2
BUN (mg/dL)	17.6±4.9
Creatinine (mg/dL)	0.9±0.2
CRP (mg/dL)	0.9 [0.7; 1.7]

Abbreviations: N, number; BMI, body mass index; CCI, Charlson's comorbidity index; SMMI, skeletal muscle mass index; TSMI, trunk muscle mass index; ASMI, appendicular muscle mass index; FVC, forced vital capacity; FEV_1, forced expiratory volume in the first second; $FEF_{25\%–75\%}$, forced mid-expiratory flow; AST, aspartate aminotransferase; ALT, alanine transaminase; BUN, blood urea nitrogen; CRP, C-reactive protein

Data are presented as mean±standard deviation for parametric continuous variables, median [first quartile; third quartile] for non-parametric continuous, and n (%) for categorical variables; *P<0.05

Figure S1. Annual changes in spirometry findings

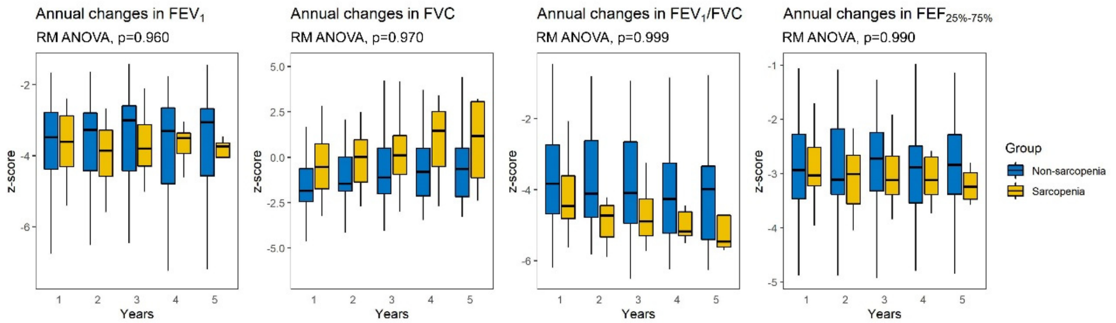

Supplement: Supplementary file 1 [file life-13-01628-s001.zip › life-2483473-supplementary.pdf]
